# Brain assembloids reveal efficacy and off target effects in high grade glioma brain cancer

**DOI:** 10.1016/j.isci.2026.117247

**Published:** 2026-08-19

**Authors:** Farhana Amy Sarker, Yuyan Chen, Victoria Prior, Sam Bax, Louise Orcheston-Findlay, Tom Grundy, Thuvarahan Jegathees, Robert Utama, Martin Engel, Tristan Rawling, Yassir Al Zubaidi, Michael Murray, Geraldine Margaret O’Neill

**Affiliations:** 1 Faculty of Medicine and Health, University of Sydney, Sydney, NSW 2006, Australia; 2 Children’s Cancer Research Unit, The Children’s Hospital at Westmead, Sydney, NSW 2145, Australia; 3 Inventia Life Science Pty Ltd., Sydney, NSW, Australia; 4 School of Mathematical and Physical Sciences, University of Technology, Sydney, NSW 2015, Australia; 5 Pharmacogenomics and Drug Development Group, Discipline of Pharmacology, School of Medical Sciences, Sydney Medical School, University of Sydney, Sydney, NSW 2006, Australia

**Keywords:** high grade glioma, anti-invasive therapy, organoid co-cultures, side effects

## Abstract

Anti-invasive therapies may limit damage to healthy brain tissue caused by invading high-grade glioma (HGG) cells. However, the actin machinery that drives invasion is shared by cancer and normal cells, and therefore, there is a risk of on-target, off-tumor side effects. We compared omega 3 polyunsaturated fatty acid epoxide analog *p*-tolyl-ureidopalmitic acid (PTU) effects between HGG multicellular tumor spheroids (MCTSs) embedded in Matrigel and assembloids consisting of HGG MCTS with embryonic stem cell-derived cortical brain organoids. PTU inhibited cofilin and induced cell rounding consistent with a loss of filamentous (F-) actin and efficiently inhibited invasion in 3D Matrigel invasion assays, but effects were cell line specific in assembloids. Immunofluorescence and GeoMx Digital Spatial Profiling of assembloids revealed that PTU disrupts signaling in cancer cells and the “healthy” cortical organoid. This study demonstrates a methodological approach for *in vitro* testing of efficacy and safety for anti-invasive therapies for HGG.

## Highlights

• Anti-Invasive drugs may arrest destructive invasion by HGG brain cancers
• Advanced preclinical models enable early efficacy and safety testing
• Anti-Invasive drug PTU differentially attenuated HGG invasion in 3D cell models
• Assembloid models allowed concomitant detection of off-tumor effects of PTU

## Graphical abstract

**Figure:**
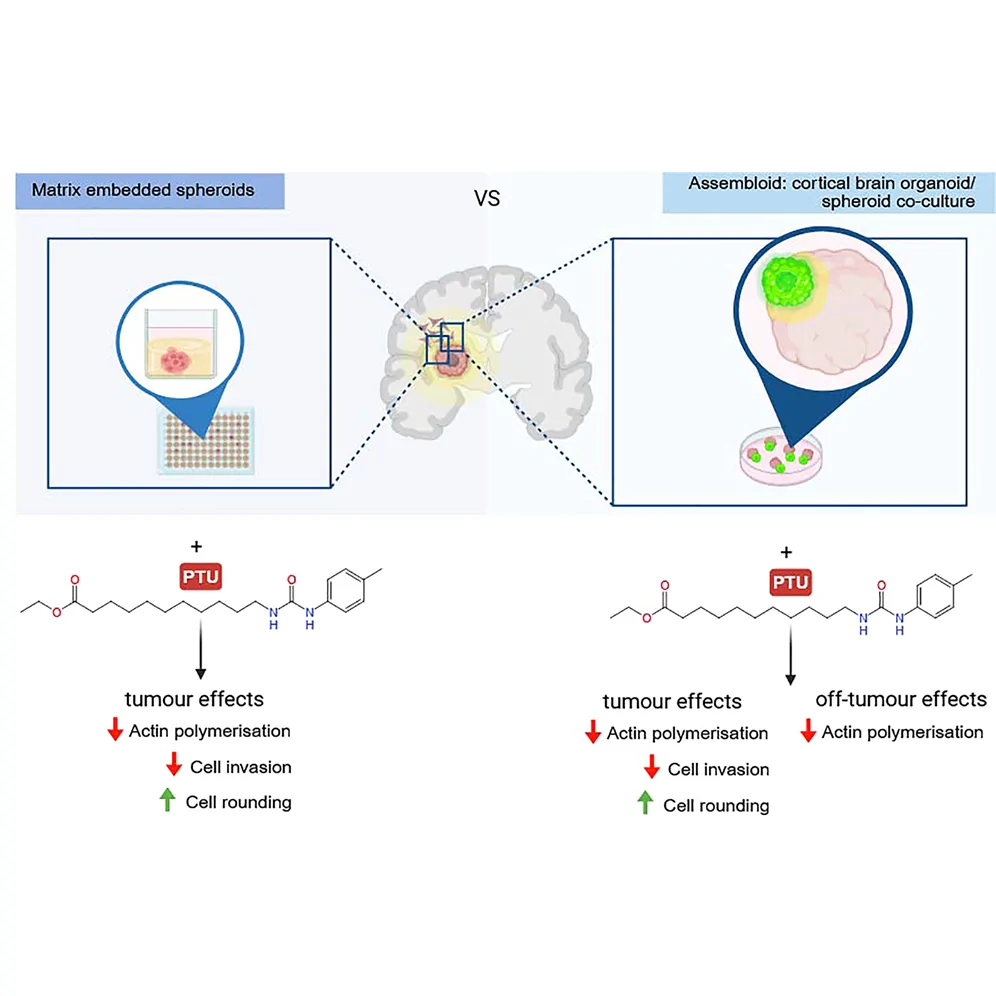

## Teaser

Natural sciences; Biological sciences; Neuroscience; Cell biology

## Introduction

High-grade glioma (HGG) brain tumors remain fatal in adults and children.^1^ Their highly disseminating nature is a major complication in establishing curative therapies, preventing complete tumor elimination through surgery and radiotherapy. Since invasion is a defining feature of HGGs, anti-invasive agents have therapeutic promise, potentially reducing tissue destruction and circumscribing invasion to thereby reduce the operative and radiotherapy treatment fields. However, new treatments for HGG have been hampered by a lack of correlation between preclinical and patient efficacy data, highlighting the need for improved preclinical models.^2^ Since it is well-established that the patterns of HGG invasion *in vivo* are determined by the underlying brain architecture,^3^ there is a requirement to mimic these features in *in vitro* assays. Furthermore, incorporation of normal brain tissue mimetics into *in vitro* assays enables concomitant assessment of off-tumor effects early in the development pipeline. In the present study we used *in vitro* assays modeling invasion to assess the anti-invasive potential of the recently described agent *p*-tolyl-ureidopalmitic acid (PTU)^4^ for HGG.

PTU is an omega-3 polyunsaturated fatty acid epoxide analog, chemically modified to resist metabolic deactivation by epoxide hydrolase enzymes, thus increasing *in vivo* stability.^4^ PTU inhibited breast cancer cell invasion through 3D Matrigel and metastasis in breast cancer mouse models.^4^ Mechanistically, PTU culminated in collapse of the filamentous (F)-actin structures^4^ that provide the force and signal integration that are required to navigate the surrounding tissue by uncoupling the cells’ ability to form strong adhesions to the matrix to generate the force required to move.^5^^,^^6^ Structural analogs of PTU did not affect breast cancer cell invasion but instead induce apoptosis.^4^ By contrast PTU was shown to be strictly anti-invasive and did not affect breast cancer cell survival. Importantly, omega-3 polyunsaturated fatty acids are readily taken up into the brain^7^ and thus represent a potential anti-invasive therapy for HGG.

The response of the HGG to unique brain architecture has long been understood as a key feature in the pathology of these malignancies.^3^ The biochemical, structural and mechanical parameters encountered as HGG disseminate throughout the brain, are integrated through the cytoskeleton to facilitate movement.^8^^,^^9^ Established routes of HGG invasion include perivascular domains and the subpial glial limiting membrane,^3^ regions that are enriched for the extracellular matrix (ECM) components fibronectin and laminin. Since the basement membrane extract Matrigel contains these ECM components, Matrigel incorporates aspects of the *in vivo* microenvironment and thus provides a convenient 3D environment for assessing HGG invasion. However, the gels lack other key components of the tumor microenvironment, and therefore, may only provide a readout for a subset of invasive behavior. Cancer cells exhibit invasion plasticity, transitioning between modes of invasion in response to cues found in the local microenvironment.^8^^,^^9^^,^^10^^,^^11^ Thus models are required that facilitate analysis of the range of invasion that characterizes *in vivo* tissue dissemination.

Another important consideration in the assessment of any agent is the potential for collateral damage of healthy tissue that is not recapitulated in simpler laboratory assays, such as Matrigel embedded MCTS. While the use of orthotopic mouse models facilitates both the investigation of invasion (e.g., Osswald et al^12^) and potential off-target effects, they are expensive and time-consuming, and there is an ethical imperative to reduce the use of animals in research. Thus, there is a requirement for intermediary *in vitro* models that facilitate analysis of cell invasion and effects on healthy tissue in early preclinical models,^13^^,^^14^^,^^15^^,^^16^ along with consideration of HGG/neuronal cell interactions, that are emerging as key determinants of HGG malignancy.^17^^,^^18^ The advent of human organoid technology now provides an opportunity to address this gap.^19^ Using defined protocols for differentiation, organoids representing different human tissues, displaying multi-lineage cell types and organized tissue architectures can be generated. Co-culture of HGG cells with stem-cell derived organoids with brain-like features provides a unique *in vitro* model platform of HGG dissemination into healthy neuronal-like tissue.^20^^,^^21^^,^^22^^,^^23^ Our recently established assembloid model, comprising multi-lineage cortically fated organoids combined with HGG multicellular tumor spheroids (MCTSs), recapitulated invasion of both pediatric diffuse intrinsic pontine glioma (DIPG) and adult glioblastoma (GBM) HGGs.^21^

In the present study we investigate the anti-invasive effects of PTU, comparing simple cultures of MCTS embedded in Matrigel with assembloids. We further demonstrate the power of the assembloids by analyzing the normal cell component represented by the cortical organoid portion of the assembloid, to assess off-target effects of PTU. Collectively, the data establish PTU is anti-invasive in patient-derived HGG cell lines in Matrigel, but also reveals differences in anti-invasive effects when applied to assembloids and identifies changes to the healthy brain tissue. The results demonstrate the importance of *in vitro* models mimicking the *in vivo* environment for the early preclinical assessment of therapeutic candidates.

## Results

### PTU slows HGG MCTS invasion in Matrigel

**Figure 1:**
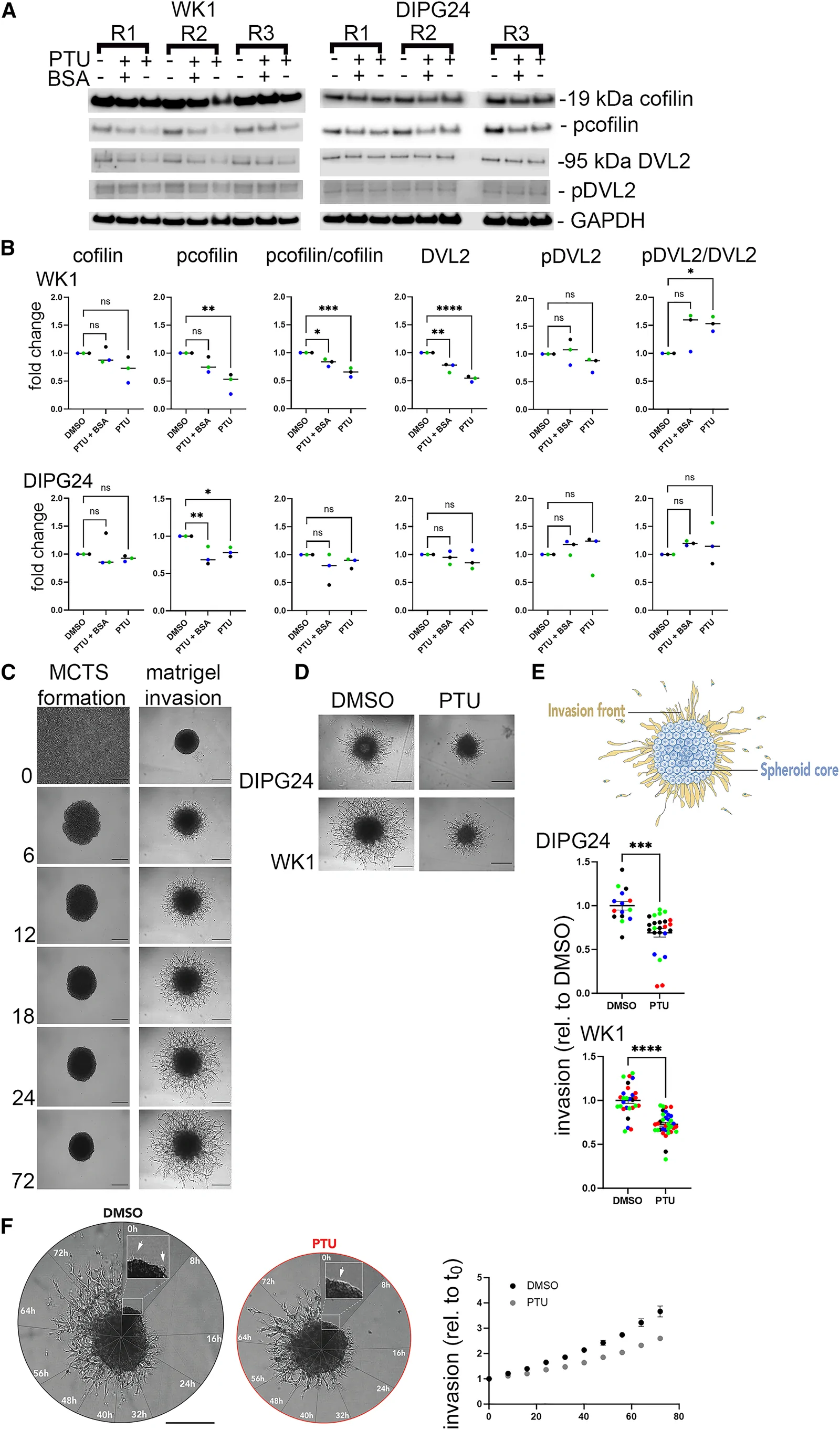
PTU inhibits MCTS invasion (A) Western blot analysis of actin-regulatory proteins cofilin, phosphorylated cofilin (pcofilin), disheveled 2 (DVL2), and phosphorylated DVL2 (pDVL2). Cells were treated with DMSO vehicle, PTU with bovine serum albumin (BSA), or PTU alone, as indicated. Extracts were prepared from triplicate repeats of WK1 and DIPG24 spheroids, as indicated (R1–R3). GAPDH was used as a loading control. Uncropped western blots are deposited in Mendeley (https://doi.org/10.17632/7tb99r5f65.1). (B) Densitometric quantification of the indicated proteins from triplicate repeats. All proteins were normalized to GAPDH and the fold change then expressed relative to DMSO control expression. ∗∗∗∗*p* < 0.0001, ∗∗∗*p* < 0.001, ∗∗*p* < 0.01, ∗*p* < 0.05, one-way ANOVA with Dunnett multiple comparisons test. (C) Bright field images of WK1 MCTS formation, showing aggregation of cells over 72 h (left), and MCTS invasion over 72 h following embedding in Matrigel (right). Scale bars, 200 μm. (D) Representative images of DIPG24 and WK1 MCTS 72 h after treatment with either 10 μM PTU or DMSO control. Scale bars, 200 μm. Note that the DMSO WK1 control image in (D) is identical to the 72 h image shown in (C) and is intentionally reproduced to provide the control comparator for the drug-treated conditions. (E) Schematic representation of image delineation for quantification of invasion area. Scatterplots show the invasion area expressed relative to the DMSO controls. Colors represent experimental repeats from different days, *n* = 4 independent repeats (green, black, blue, and red) with each data point representing an individual MCTS. Bars show the mean ± SEM. ∗∗∗*p* < 0.001, ∗∗∗∗*p* < 0.0001, students’ *t* test. (F) Representative time-lapse images arranged as a radial time series, with each wedge representing successive 8-h intervals of WK1 MCTS. Arrows (insets) indicate extension of cells into the matrix in the earliest period. Graph shows the area of invasion relative to the matched condition at the start of imaging (t_0_). *n* = 4 independent repeats and *n* ≥ 3 spheroids analyzed per condition per repeat. Data points show mean ± SEM. Scale bars, 200 μm.

PTU was reported to inhibit Rho GTPase signaling, reducing activation of the RhoGTPase regulatory factor disheveled 2 (DVL2) and inhibiting the downstream actin-severing protein cofilin.^4^ We first assessed the effect of PTU on this signaling pathway, comparing effects in the DIPG line DIPG24 and the GBM line WK1. To better maintain the phenotype and genotype of the primary tumor source,^24^ these cell lines are cultured in serum-free media supplemented with growth factors. Due to the low solubility of PTU and fetal bovine serum (FBS), we compared PTU effects in the presence and absence of bovine serum albumin (BSA) to potentially improve the drug effects. Analysis of protein extracts from MCTS treated with PTU reveals that cofilin phosphorylation was significantly reduced in both WK1 and DIPG24 MCTS treated with PTU (*p*-cofilin, Figures 1A and 1B). Agreeing with the earlier reports, DVL2 expression was significantly reduced in WK1 spheroids; however, levels were unaffected in the DIPG24 cells (Figures 1A and 1B). In each case PTU effects were stronger in the absence of BSA (Figures 1A and 1B), therefore BSA was not used in subsequent experiments. Collectively, the data demonstrate that cofilin activity is decreased by PTU treatment in the HGG lines under investigation.

Next, we assessed the effect of PTU on HGG invasion in MCTS embedded in Matrigel. Both DIPG24 and WK1 cells collectively invaded^25^ into the Matrigel matrix (Figures 1C and 1D). PTU addition significantly reduced invasion of each line to a similar extent (Figures 1D and 1E). Time-lapse images (Figure 1F) demonstrate that the anti-invasive effects of PTU were maintained over the time course of exposure. At the earliest time point, fewer cell membrane protrusions from PTU-treated WK1 are observed extending into the matrix (Figure 1E, insets arrows). Subsequently, the rate of invasion from the PTU-treated MCTS is reduced. To explore whether additional environmental parameters influence invasion and therapeutic response, we also performed a pilot bioprinting experiment varying matrix stiffness suggesting that matrix stiffness has a limited effect on PTU efficacy (Figure S1).

**Figure 2:**
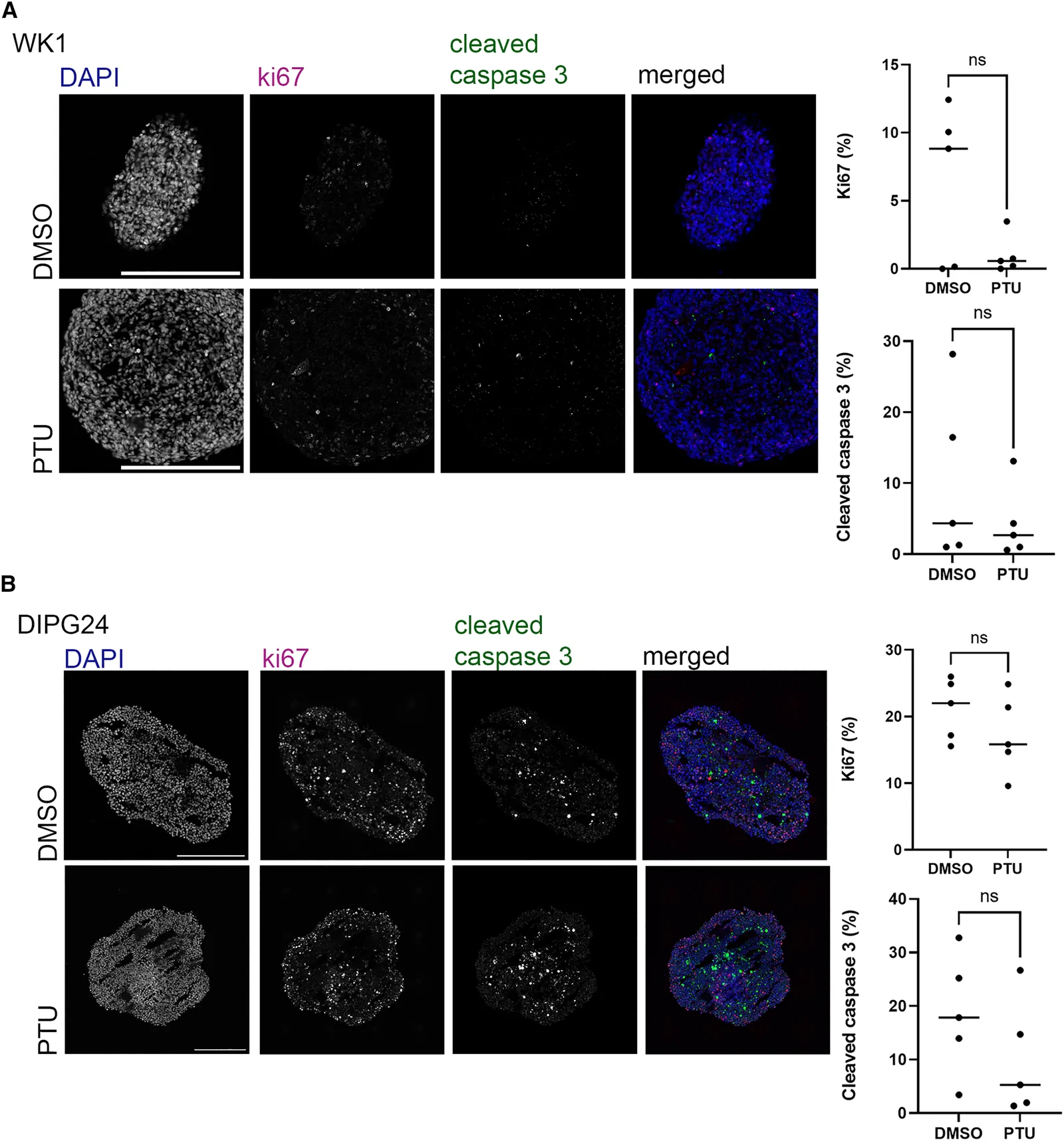
PTU does not affect cell proliferation or apoptosis (A) Cryo-sections of OCT embedded WK1 MCTS, immunostained with DAPI to identify nuclei, Ki67 to detect proliferating cells and cleaved caspase 3 to detect apoptotic cells. Scale bars, 250 μm. Graphs to the right depict quantification of the percentage of Ki67 and cleaved caspase 3 positive cells. *N* = 5 independent MCTS per cell line, per condition. (B) DIPG24 MCTS analyzed as described in (A). N = 5 independent MCTS per cell line, per condition. NS = not significant, students’ *t* test.

Given that changes in proliferation may also affect apparent invasion results, we tested the effect of PTU on cell proliferation in WK1 and DIPG24 MCTS. Cultures were fixed and immunostained for the proliferation marker Ki67 and the apoptosis marker cleaved caspase 3 (Figures 2A and 2B). Quantification revealed that there was no significant difference in either proliferation or apoptosis in the presence of PTU (Figures 2A and 2B). This aligns with previous reports demonstrating that PTU inhibits invasion but does not inhibit proliferation in breast cancer cells.^4^

### PTU reduces invasion into cortical brain organoids

**Figure 3:**
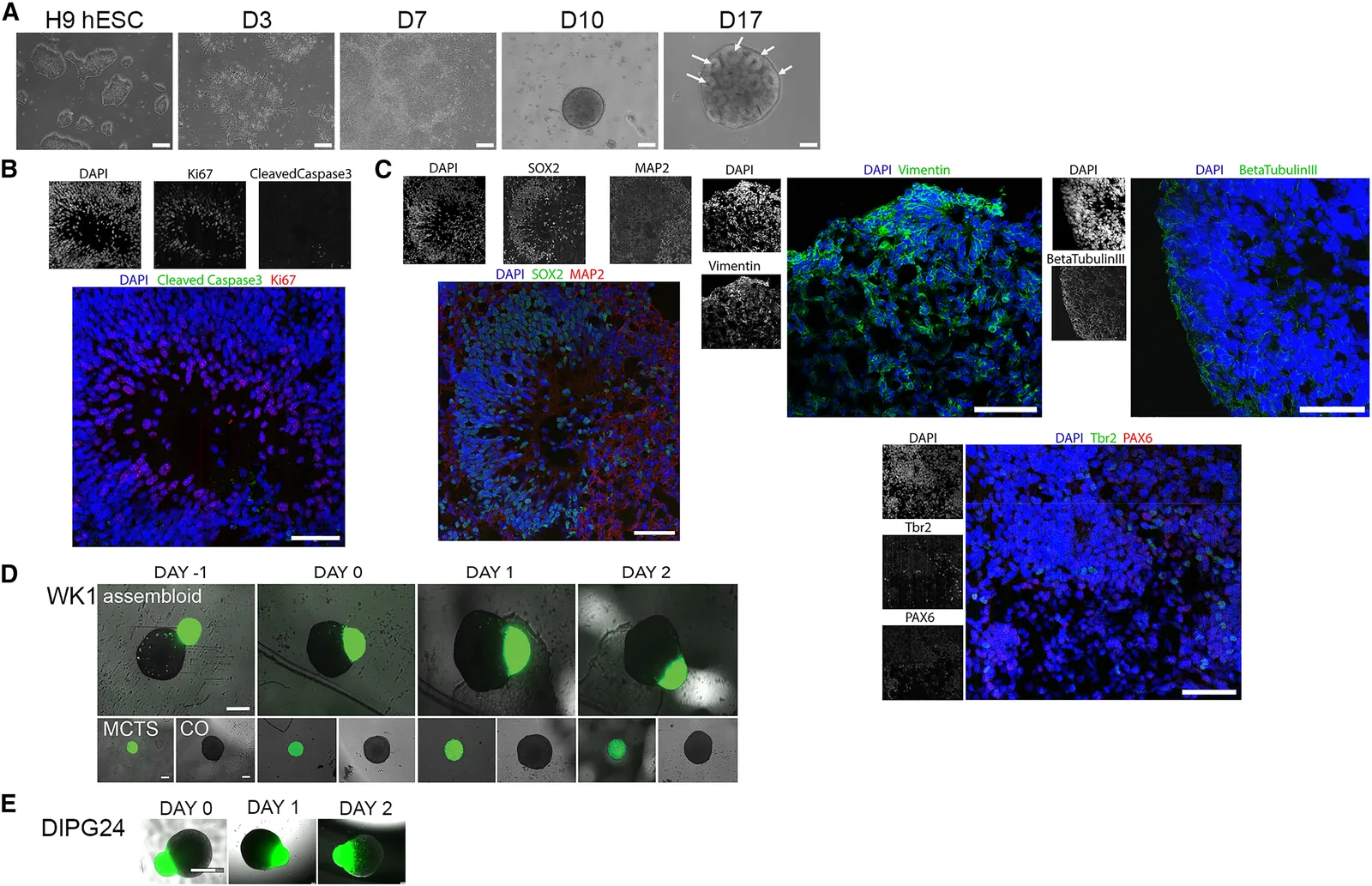
Generation of assembloids (A) Brightfield images showing neural induction and cortical organoid generation. H9 cells are cultured on vitronectin-coated dishes, then passaged onto laminin-coated plates and induced to neural fate with dual SMAD inhibitors. Colonies show signs of differentiation at day 3, with distinct individual cells at the periphery of cell colonies. On day 7, layers of ectodermal-fated cells are harvested and transferred to suspension culture. Harvested neural progenitor cells aggregate into spheres under low attachment conditions (day 10). As organoids increase in size, neural rosettes (white arrows, day 17) are apparent. Scale bars, 50 μm. (B) Representative images of a cortical organoid neural rosette immunostained for the proliferation marker Ki67 (red) and apoptosis marker cleaved caspase 3 (green). Scale bars, 50 μm. (C) Representative images showing a neural rosette stained for radial glial progenitor cells (SOX2, green) and surrounding neuronal dendrites (MAP2, red), as well as markers for ventricular zone progenitors (PAX6, vimentin), immature neurons (β-Tubulin III) and intermediate progenitors (Tbr2). Scale bars, 50 μm. (D) Representative images of WK1 assembloids. Day 1 denotes day of assembloid initiation. WK1 cells labeled with CellTracker Green (CTG). Top section shows assembloid cultures over the indicated time course and bottom images show control MCTS and cortical organoids (COs) cultured alone under the indicated conditions. Scale bars, 200 μm. (E) Representative images of DIPG24 assembloids forming over the indicated time course. DIPG24 cells tagged with GFP. Scale bar, 728 μm.

While the Matrigel invasion data provide a readout for invasive modes that are specific to the Matrigel environment, this does not capture the complexity of the brain tumor environment. Therefore, we investigated whether PTU similarly inhibits HGG invasion in a more brain-like tissue environment. To address this question, we applied the brain assembloid model that we have developed.^21^ To generate the assembloids, cortical organoids were prepared following our established protocols. Directed differentiation of H9 hESC resulted in the formation of neural rosettes characteristic of mature cortical organoids (Figure 3A). Proliferation within the neural rosettes was confirmed by Ki67 immunostaining, with little evidence of apoptosis as detected by cleaved caspase 3 (Figure 3B). The presence of multiple cell lineages in the organoids was demonstrated through immunostaining for radial glial progenitor cells, immature neurons, ventricular zone progenitors and intermediate progenitors (Figure 3C). Assembloids were then generated from preformed organoids by combining either with cell tracker green (CTG)-labeled WK1 MCTS (Figure 3D) or GFP-tagged DIPG24 MCTS (Figure 3E).

**Figure 4:**
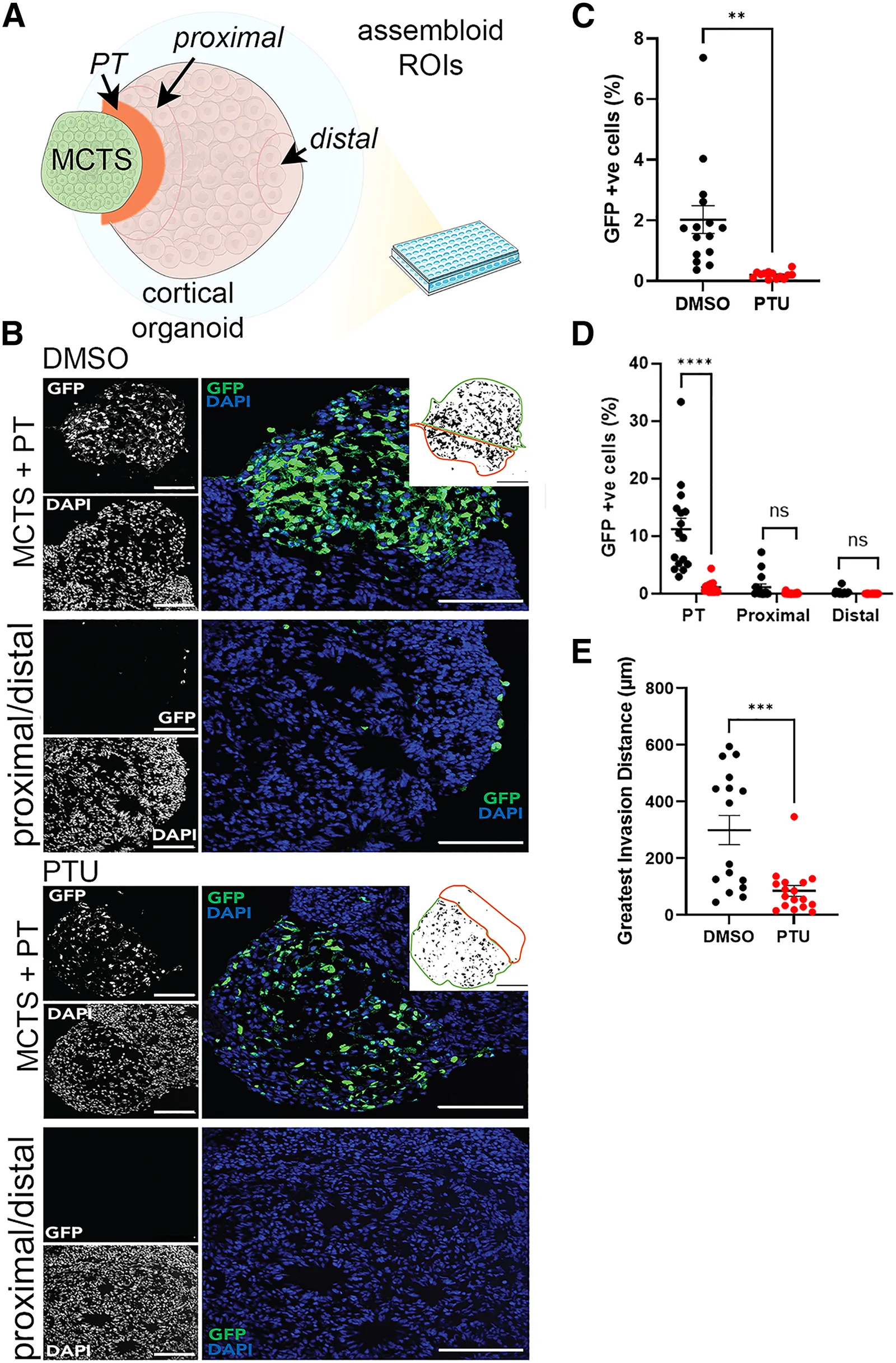
PTU inhibits WK1 invasion into cortical organoids (A) Schematic depiction of the segmented ROIs for invasion analyses including the MCTS, peri-tumoral zone (PT), the proximal region of the CO and the distal region of the CO. As indicated in the diagram assembloids were prepared and incubated in 96-well dishes. (B) Representative maximum intensity z-projections of confocal images of WK1 assembloids treated as indicated, showing the regions encompassing the MCTS and PT (top) and the proximal/distal organoid (bottom). Pairs of images (MCTS + PT and proximal/distal), were acquired from the same organoid. In each case, the fields of view shown are spatially adjacent and partially overlap. Images show GFP-positive WK1 cells, DAPI stained-nuclei and merged images. Insets show binary masks of GFP-positive WK1 cells within the MCTS (green outline) and the PT (orange outline). Scale bars, 200 μm. (C) Proportion of total WK1 cells that have invaded, expressed as percentage of the total cells in the MCTS. Each dot point represents an individual assembloid culture, (DMSO black *n* = 15, PTU red, *n* = 13). (D) Proportion of GFP-positive WK1 cells in each indicated ROI (PT, proximal, distal), expressed as percentage of the total GFP-positive WK1 cells. Each dot point represents an individual assembloid culture, (DMSO black *n* = 16, PTU red, *n* = 17). (E) Data showing the furthest total distance (μm) of WK1 cells measured from the MCTS border. Error bars show the SEM. Each dot point represents an individual assembloid culture, (DMSO black *n* = 16, PTU red, *n* = 17). NS = not significant, ∗*p* < 0.05, ∗∗*p* < 0.01, ∗∗∗*p* < 0.001, ∗∗∗∗*p* < 0.0001, students’ *t* test, except for (D) where data were compared by two-way ANOVA with Tukey post-comparison test.

To quantify PTU effects on WK1 invasion in the brain-like environment, assembloids incorporating WK1 MCTS were imaged and maximal z-projections were then demarcated into regions of interest (ROIs) including the MCTS, the PT, proximal and distal regions (Figure 4A). Under control conditions WK1 cells diffusely invaded into the organoid (Figure 4B). Following PTU treatment, significantly fewer cells invaded (Figure 4C). Under control conditions the greatest proportion of invasive WK1 cells was seen in the PT region, which was attenuated by PTU (Figure 4D). Finally, PTU reduced the maximum distanced invaded by any WK1 cell (Figure 4E).

**Figure 5:**
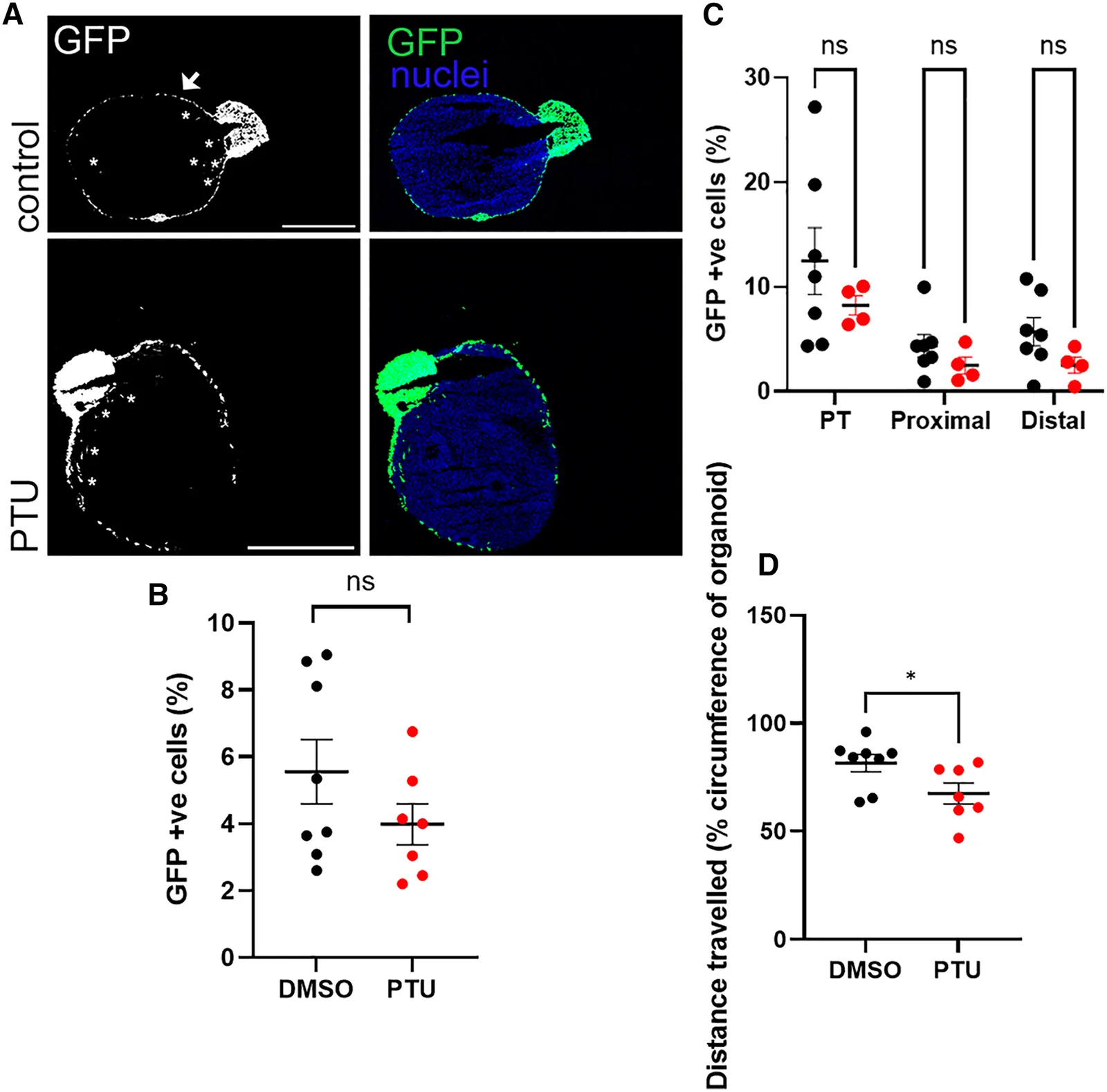
PTU effects on DIPG24 invasion in assembloids (A) Representative maximum intensity z-projections of confocal images of DIPG24 assembloids treated as indicated. Images show GFP-positive DIPG24 cells and GFP images merged with corresponding DAPI-stained nuclei. Asterisks denote DIPG24 cells that have invaded into the organoid portion of the assembloid. Arrow denotes DIPG24 cells migrating along the periphery of the organoid. Scale bars, 500 μm. (B) Proportion of total DIPG24 cells that have invaded, expressed as percentage of the total cells in the MCTS. Each dot point represents an individual assembloid culture, (DMSO black, *n* = 8, PTU red, *n* = 7). (C) Proportion of GFP-positive DIPG24 cells in each indicated ROI (PT, proximal, distal), expressed as percentage of the total GFP-positive DIPG24 cells. Each dot point represents an individual assembloid culture, (DMSO black, *n* = 8, PTU red, *n* = 4). (D) Graph shows the percentage of the organoid circumference positive for DIPG24 cells. Each dot point represents an individual assembloid culture, (DMSO black, *n* = 8, PTU red, *n* = 7). Error bars show the SEM. NS = not significant, ∗*p* < 0.05, students’ *t* test, except for (C) where data were compared by two-way ANOVA with Tukey post-comparison test.

We next assessed PTU effects on DIPG24 invasion in assembloids. As we have reported previously,^21^ the DIPG24 cells invaded into the cortical organoid (Figure 5A, asterisks) and along the outer-surface of the organoid (Figure 5A, arrow). Observation of the DIPG24 assembloids after PTU treatment, suggested a similar pattern of invasion, with cells observed to invade into the cortical organoid and to migrate along the outer surface. In this case, there was little detectable effect on the cells that invaded into the organoid; however, the cells invading along the surface of the organoid did not appear to reach the opposite end of the organoid (Figure 5A). Quantification confirmed no significant difference in the total percentage of DIPG24 cells that invaded into and along the cortical organoid (Figure 5B) and correspondingly, no difference in infiltration into the different ROIs (Figure 5C). Conversely, calculation of the total distance traveled around the circumference of the organoid revealed a small but significant decrease in the presence of PTU (Figure 5D). Thus, PTU reduced DIPG24 migration along the organoid surface but did not affect invasion into the organoid.

### PTU induces cytoskeletal reorganization in 3D cultures

**Figure 6:**
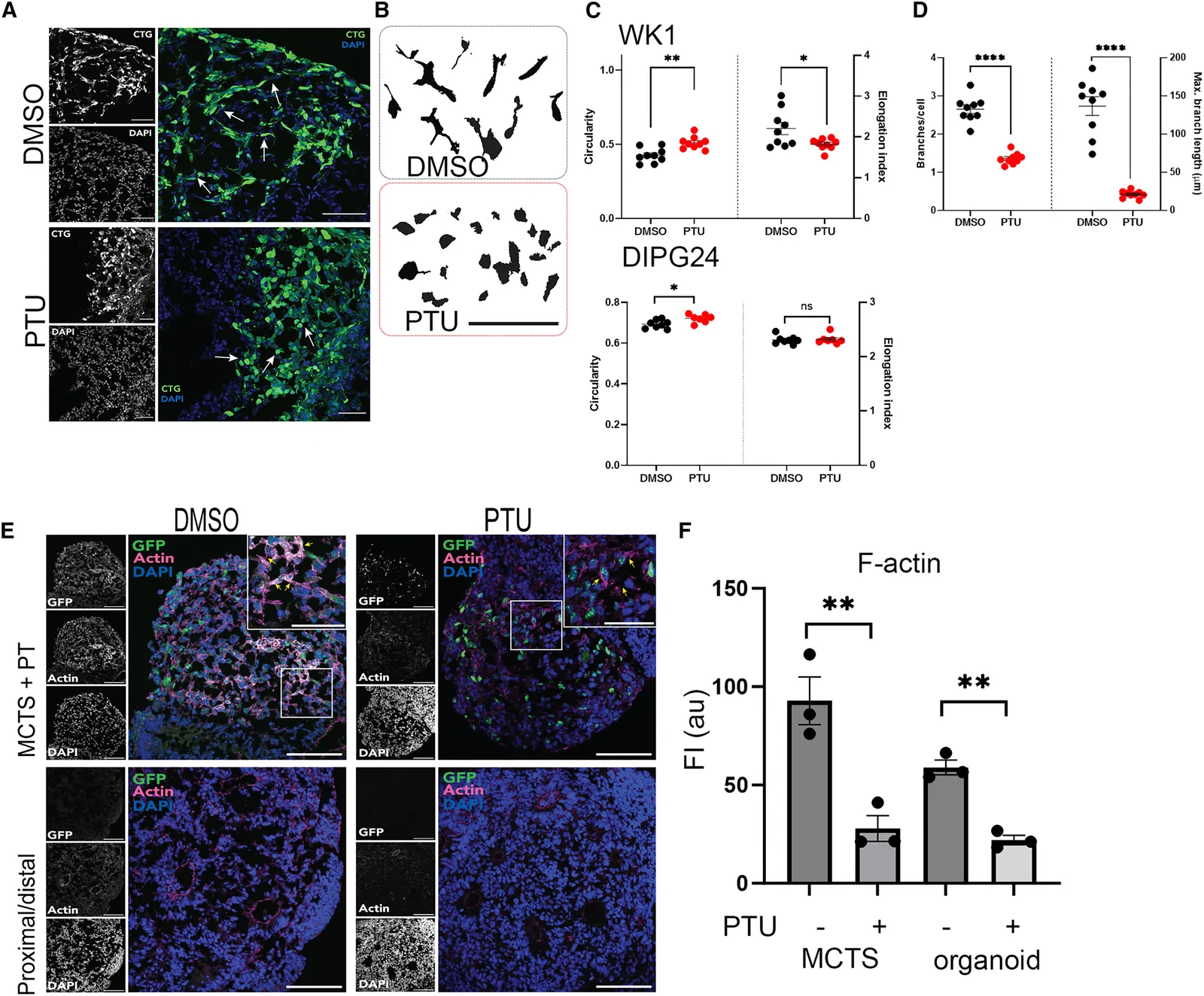
PTU decreases cytoskeletal organization (A) Maximum z-projections of confocal sections of WK1 assembloids. WK1 cells are labeled with CellTracker green and cell nuclei are counterstained with DAPI (blue). Arrows highlight examples of elongated cells under control conditions (DMSO) and rounded cells in PTU treated cultures, respectively. Scale bars, 100 μm. (B) Exemplar binarized segmented cells from the indicated treatment conditions. Scale bars, 100 μm. (C) Circularity ratio (left axis) and elongation index (right axis) for WK1 and DIPG24 cells from assembloid cultures, under the indicated conditions. Morphometric analyses were performed on segmented and binarized images (*n* > 750 cells per condition). Horizontal bars show the mean and error bars = SEM. ∗*p* < 0.05, ∗∗*p* < 0.01, students’ *t* test. Dots represent averaged data from individual assembloids (control: black, PTU: red), minimum 8 assembloids per condition. (D) Number of cell projections (branches) per cell (left axis) and maximum length of individual branches (right axis). Dots represent averaged data from individual assembloids (control: black, PTU: red), minimum 8 assembloids per condition. (E) Maximum z-projections of confocal sections of assembloids showing the MCTS + PT portion of the assembloid and the distal organoid section as labeled and as per the schematic in Figure 4A. Pairs of images (MCTS + PT and proximal/distal) were acquired from the same organoid. WK1 cells are tagged with GFP and counter-stained with phalloidin to detect filamentous actin (F-actin, magenta). Scale bars, 100 μm, except for scale bars in merged color insets which = 50 μm. Yellow arrows in insets highlight F-actin structures. (F) Graph shows the average fluorescence intensity (FI) in arbitrary units (aus) for F-actin under control versus PTU-treated conditions and in the MCTS versus the organoid portion of the assembloid. Error bars = SEM. ∗∗*p* < 0.01, students’ *t* test, *n* = 3 independent assembloids.

WK1 cells appeared more spherical after PTU treatment in the assembloids (Figures 6A and 6B). Quantification confirmed that WK1 cells were rounder, with fewer and shorter membrane protrusions per cell after PTU treatment (Figure 6C). Therefore, we also questioned the morphology of the DIPG24 cells post-PTU treatment. As was seen for the WK1 assembloids, PTU induced DIPG24 rounding (Figure 6C). The DIPG24 cells were rounder in the assembloid cultures under control conditions than the WK1 cells (Figure 6C) and did not display branched processes.

The cell rounding phenotype induced by PTU aligns with earlier reports that PTU suppresses actin dynamics that regulate cell morphology^4^ and our data showing loss of cofilin expression (Figures 1A and 1B). To visualize PTU effects on actin organization, we next assessed filamentous (F)-actin by immunostaining with fluorescently tagged phalloidin. Importantly, phalloidin can only bind to polymerized actin, therefore loss of actin staining reflects loss of F-actin. Phalloidin staining in PTU treated WK1 assembloids appeared reduced in the MCTS-portion of the assembloid (Figure 6E) and quantification confirmed significantly reduced phalloidin staining (Figure 6F). We also observed significantly reduced phalloidin staining in the organoid component of the assembloids (Figure 6F). This thus suggested that in addition to affecting the tumor cells, PTU has potential on-target, off-tumor effects in healthy tissue, reducing F-actin in the healthy organoid tissue.

**Figure 7:**
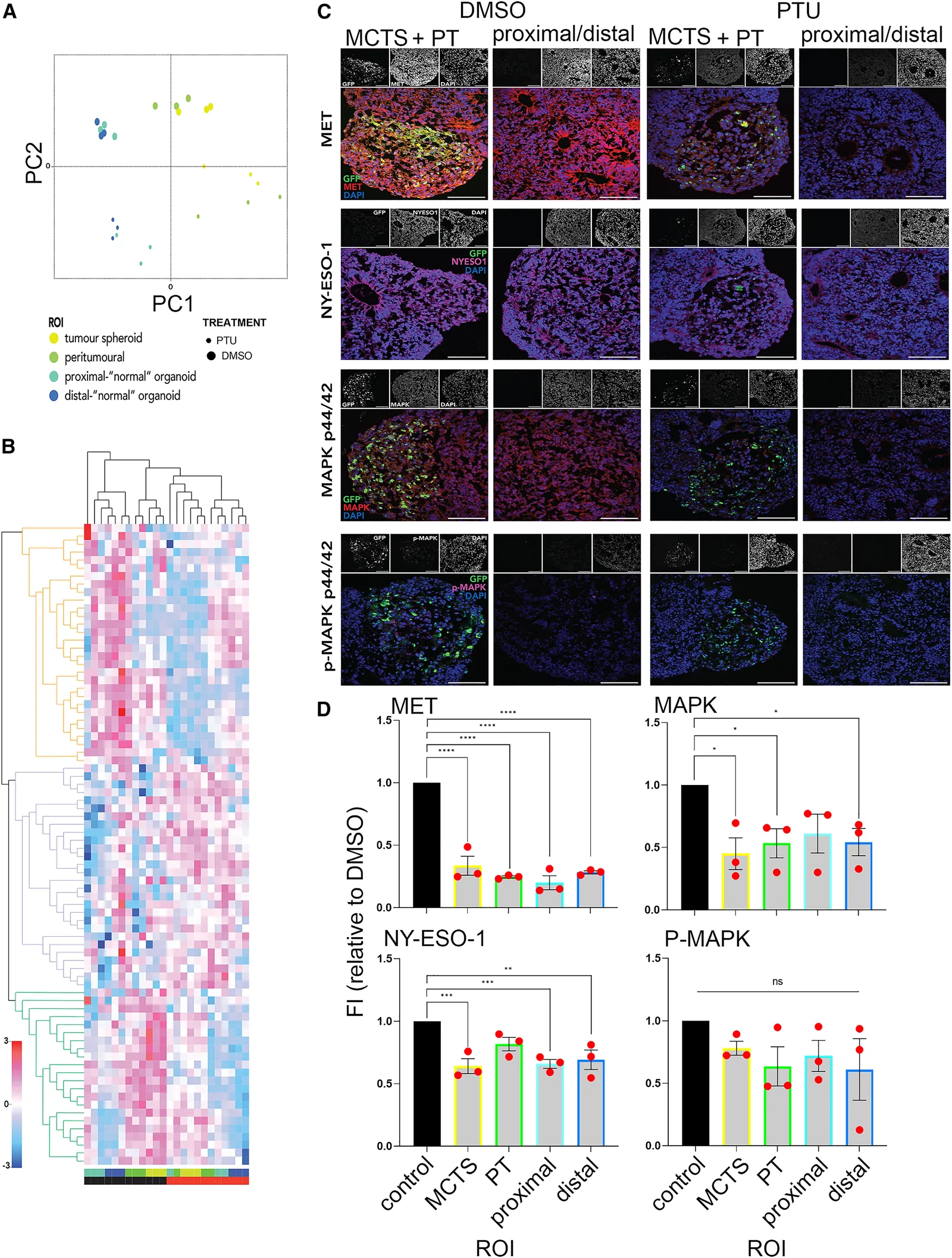
PTU effects signaling in cortical organoids (A) PCA reveals grouping of protein count data with ROI and treatment status (large circles denote DMSO, small circles denote PTU-treated samples). Data represent 3 assembloid replicates per treatment condition. (B) Heatmap of normalized protein expression from control assembloids (black bar at bottom of figure) versus PTU-treated assembloids (red bar at bottom of figure). Hierarchical protein clustering is based on similarities of target proteins and is delineated by adjacent colored dendrograms. Further grouping into ROIs is indicated by the key along the *x* axis. Look-up table indicates *Z* score, where increasing values are indicated by a shift from blue to red. (C) Representative maximum z-projections (*n* > 3 independent repeats per condition) of confocal images cryosections of WK1 assembloids immunostained for expression of MET (red), NY-ESO-1 (magenta), MAPK p44/42 (red) and *p*-MAPK 44/42 (magenta). WK1 cells are tagged with GFP. MCTS + PT and distal organoid regions of the assembloids are shown in the left and right columns, respectively and as per the schematic shown in Figure 4A. Pairs of images (MCTS + PT and proximal/distal) were acquired from the same organoid. In some cases (NY-ESO-1), the fields of view shown are spatially adjacent and partially overlap. Insets show gray scale images for each channel shown in the larger color merged image. Scale bars, 100 μm. (D) Graphs show fluorescence intensity (normalized to area of ROI) and expressed relative to the matched DMSO control ROI for the indicated proteins. ROIs are indicated on the *x* axis of the bottom 2 graphs. Points show the average value from 3 independent experiments. Error bars = SEM. ∗*p* < 0.05, ∗∗*p* < 0.005, ∗∗∗*p* < 0.0005, NS = not significant, two-way ANOVA with Tukey’s post-comparison test.

To further explore effects of PTU on non-tumor cells, WK1 assembloids were profiled using the GeoMx digital spatial profiling (DSP) platform. The human immuno-oncology panel was selected as the comprehensive representation of tumors and the tumor microenvironment. This panel covers 7 signaling modules, including the Profiling Core (a series of cell markers and controls), immune-oncology drug targets, immune activation status, pan-tumor, cell death, PI3K/AKT signaling and MAPK signaling. Principal component analysis (PCA) of the GeoMx data showed that together PC1 and PC2 accounted for 51% of the data variance. PC1 separated samples based on ROI, separating organoid-specific ROIs (proximal and distal) from tumor cell-associated ROIs (spheroid and PT) (Figure 7A). PC2 separated samples based on PTU treatment, including showing distinct expression between PTU-treated and control organoid, suggesting that PTU also altered signaling in the cortical organoid cells. A hierarchal dendrogram heatmap was generated within the GeoMx DSP analysis suite from the normalized datasets to identify the underpinning PC clusters (Figure 7B). Three expression clusters were apparent (orange, purple, and green branching; Figure 7B), representing predominant groupings including the PI3K/AKT (orange cluster), the immune-oncology drug target and immune activation status (purple cluster) and the pan tumor and cell death panel (green cluster). PI3K/AKT associated markers (Akt, phospho-Akt [S437], MET, phospho-GSK3B [S9], phospho-GSK3A [S21]/phospho-GSK3B, phospho-PRAS40 [T246], and phospho-tuberin [T1462]) were most notably decreased in tumor regions of PTU-treated co-cultures (orange cluster, Figure 7B). Overall, there was a trend for reduced expression of the pan tumor cell death panel markers in the presence of PTU (BCL-2, EpCAM, HER2/ERBB2, PTEN, BCLXL, BIM, and p53), but this was most apparent in the proximal and distal organoid ROIs (green cluster, Figure 7B). Finally, there was trend for increased expression of marker proteins from the immune-oncology drug target and immune activation status (ARG1, LAG3, CD25, and CD27) in the presence of PTU, across all ROIs (purple cluster, Figure 7B).

Next, expression of NY-ESO-1, MET, total p44/42 MAPK (tMAPK) and phosphorylated p44/42 MAPK (*p*-MAPK) were independently analyzed. These selections were based on differential expression between ROIs in the GeoMx data and further stratifications based on general cancer signaling and cell migration terms resulting from enrichment analysis (Figures S2 and S3). Detection was significantly decreased for each protein across all ROIs examined in the presence of PTU, except for *p*-MAPK (Figures 7C and 7D). Thus, in addition to significantly decreased F-actin (Figure 6F), PTU appeared to modulate other signaling pathways in cortical organoids. In conclusion, while PTU inhibited HGG invasion, we also identified potential changes to normal tissue, highlighting the value of incorporating the healthy tissue mimetic in preclinical assessments.

## Discussion

A lack of correlation between preclinical assessments and clinical efficacy in the development of new drugs for HGG significantly hampers efforts to improve patient survival.^2^ To address this deficiency, *in vitro* assessments incorporating critical aspects of the brain tumor microenvironment were used in the present study. The benefits of this approach are 2-fold. Firstly, the models provide *in vivo* like tissue structure and organization, facilitating analysis of HGG invasion. Secondly, analysis of HGG cells in the context of healthy neuronal cells in the form of cortical organoids facilitated concomitant analysis of potential side effects on healthy tissue.

Congruent with the previous report that PTU affects the actin cytoskeleton through inhibition of DVL2 and cofilin activation,^4^ we find that activity of both proteins is reduced in PTU-treated WK1 MCTS. However, only cofilin activity was affected in DIPG24 MCTS. Since PTU significantly reduced invasion of both WK1 and DIPG24 MCTS in Matrigel invasion assays, this suggests that PTU does not inhibit invasion of these cell lines via a DVL2-dependent mechanism. Instead, the finding that cofilin is significantly down-regulated in both lines following PTU treatment, suggests a potential role for cofilin in PTU-mediated inhibition of Matrigel invasion.

The Matrigel invasion assays provide a limited replication of the brain microenvironment, constituting a purified matrix that lacks key features of the *in vivo* brain structure and composition. Acknowledging this, we also performed exploratory bioprinting experiments to assess the impact of matrix stiffness, which suggested that mechanical properties may further modulate invasion and treatment response (Figure S1). However, these findings require validation across additional HGG cell lines. Each of these approaches represents a reductionist model, allowing the analysis of individual parameters. In recognition that the brain microenvironment is a critical regulator of the characteristic invasive behavior of HGG cells there is concerted effort to derive improved model systems for testing new therapies.^13^^,^^16^ Approaches that incorporate interactions between the tumor cells and the surrounding healthy neuronal cells are key. Given the critical role of the two-way interaction between HGG cells and neuronal cells in HGG development and response to therapy,^26^ assembloids encompassing brain-mimetic organoids provide an *in vitro* method for testing HGG therapies in the context of a more complex healthy brain-like tissue.^20^^,^^21^^,^^27^^,^^28^

We show differences between the effects of PTU tested in a simple 3D Matrigel invasion assay versus the more complex assembloid model that recapitulates a brain-like tissue environment. While PTU was equally effective inhibiting invasion of both WK1 and DIPG24 lines in Matrigel, the same was not true in the assembloids. Invasion plasticity, reflected by the adoption of alternative cell morphologies, is regulated by the structural, biochemical and physical features of the tissue through which the cell is traversing.^11^^,^^29^ Under control conditions the DIPG24 cells exhibited a more rounded morphology in the assembloids, reminiscent of amoeboid-type invasion. While the WK1 cells had an elongated phenotype with extended, branched membrane processes, aligning with a mesenchymal invasion morphology. F-actin plays distinct roles in these two invasive behaviors. In mesenchymal invasion, F-actin supports strong integrin-mediated adhesive force to the matrix required for translocating the cell body.^11^ Conversely F-actin provides a squeezing force from the cell rear that is required for the path-seeking, reduced adhesion, mechanism of amoeboid invasion. The different response seen to PTU between the WK1 and DIPG24 cells in the assembloids may therefore reflect a predominantly mesenchymal mode of invasion in the WK1 cells that is susceptible to PTU inhibition. Rounded, amoeboid invasion adopted by the DIPG24 cells in the assembloid context appeared less affected by PTU-mediated disruption of actin. The Matrigel environment induced both cell lines to exhibit mesenchymal-like collective invasion. If PTU specifically inhibits mesenchymal invasion, this potentially explains why PTU effects were similar for both cell lines in the Matrigel invasion assay. Collectively, these data highlight the importance of mimicking the invasion microenvironment to capture the range of invasive behaviors and hence response to anti-invasive therapies.

In addition to revealing differences dependent on invasion plasticity, incorporation of the cortical organoid brain-like tissue accelerated the identification of potential off-tumor drug effects. Reflecting the inhibition of actin-regulators that was seen in the tumor cells, F-actin was decreased in the cortical organoid portion of the assembloids. Further exploring the off-tumor effects using spatial profiling we demonstrated downregulation of key molecules in the organoids including MET, MAPK, and NY-ESO-1. Collectively, these data suggest the potential for off-tumor effects of PTU, although the likely deleterious effects of this need further examination. MAPK suppression in non-target tissues is currently clinically acceptable; combined dabrafenib (targeting *BRAFV600*) and trametinib (MEK kinase inhibitor) is used for sustained tumor control via downstream MAPK inhibition in *BRAFV600* mutant tumors.^30^ Similarly, MET is a safe target in lung cancer,^31^ suggesting MET suppression may not result in unacceptable off-tumor effects and PTU was well-tolerated in mouse studies.^4^

The low solubility of PTU, along with the potential effects on the actin cytoskeleton, and the potential for HGG cells to evade PTU inhibition through adaptive invasion mechanisms, mean that PTU has promise for HGG, but more work is required for this drug to transition to clinical trial. The work presented provides a case study supporting the inclusion of cell models that better mimic the relevant tumor microenvironment early in the assessment of emerging therapeutics. Despite the fundamental role played by surrounding tissue in regulating cancer cell invasion and signaling,^25^^,^^32^^,^^33^^,^^34^^,^^35^^,^^36^ most *in vitro* assays lack this critical component. Consequently, tumor behavior and therapeutic response observed in simplified systems often fail to translate to the clinic. Assembloids replicate the hallmark invasive features of HGG biology and thus represent an important platform to assess therapy response and off-tumor effects early in the drug testing pipeline.

### Limitations of the study

Limitations of the study include the use of a restricted panel for the spatial signaling analysis. The study was not designed to investigate sex-specific differences in treatment response, and the limited number of patient-derived cell lines precludes meaningful assessment of sex-associated effects. Additionally, cortical organoids represent early embryonic stage brains,^37^ lack vasculature and other elements of the tumor microenvironment such as the immune cell component. Nonetheless, they provide a tissue-like mimetic for analyzing 3D invasion. In summary, our study demonstrates the value of including the brain microenvironment for assessing HGG invasion, and, concomitant assessment on non-cancer cells.

## Resource availability

### Lead contact

Requests for further information and resources should be directed to and will be fulfilled by the lead contact, Geraldine Margaret O’Neill (geraldine.oneill@sydney.edu.au).

### Materials availability

This study did not generate new unique reagents

### Data and code availability

• No high-throughput sequencing data were generated in this study. All data supporting the findings of this study are available from the corresponding author upon reasonable request.
• Original western blot images have been deposited at Mendeley at https://doi.org/10.17632/7tb99r5f65.1 and are publicly available. Microscopy data reported in this study will be shared by the lead contact upon request.
• This study does not report original code
• Any additional information required to reanalyze the reported data is available from the lead contact upon request.

## Acknowledgments

We acknowledge Dr. David Scoville and Dr Marshall Feterl from NanoString Technologies for their assistance with protein analysis and the GeoMx DSP platform. This work was supported by University of Sydney Post-graduate Training Awards (F.A.S. and V.P.); Multiomics Digital Spatial Profiling (DSP): Westmead Technology Access Grant Program (F.A.S.); 10.13039/501100018675The Kids’ Cancer Project grant; Perpetual Impact philanthropy grant; and the Balance Foundation.

## Author contributions

Conceptualization, F.A.S., Y.C., V.P., and G.M.O.’N.; formal analysis, F.A.S., Y.C., T.G., R.U., M.E., M.M., and G.M.O.’N.; funding acquisition, F.A.S., V.P., and G.M.O.’N.; investigation, F.A.S., Y.C., V.P., S.B., L.O.-F., T.G., T.J., and Y.A.Z.; methodology, R.U., M.E., T.R., Y.A.Z., and M.M.; supervision, M.E., M.M., G.M.O.’N.; writing – original draft, F.A.S. and G.M.O.’N.; writing – review and editing, F.A.S., Y.C., V.P., S.B., L.O.-F., T.G., T.J., M.E., T.R., M.M., and G.M.O.’N.

## Declaration of interests

The authors declare no competing interests.

## STAR★Methods

### Key resources table

**Table:** 

REAGENT or RESOURCE	SOURCE	IDENTIFIER
**Antibodies**		

AlexaFluor®488- conjugated Donkey anti-mouse IgG (H + L) ReadyProbes secondary antibody	Thermo Fisher Scientific	Cat#R37114; RRID: AB_2556542
AlexaFluor®488- conjugated Donkey anti-rabbit IgG (H + L) ReadyProbes secondary antibody	Thermo Fisher Scientific	Cat#R37118; RRID: AB_2556546
Anti-Dishevelled 2 (phospho S143) [EPNCIR145] Rabbit Monoclonal Antibody	Abcam	Cat#ab124933; RRID: AB_10972486
Cofilin (D3F9) Rabbit Monoclonal Antibody	Cell Signaling Technology	Cat#5175; RRID: AB_10622000
Cy3-conjugated Donkey anti-mouse IgG secondary antibody species adsorbed	Sigma-Aldrich	Cat#AP192C; RRID: AB_92642
Cy5-conjugated Cy™5 AffiniPure Donkey Anti-rabbit	Jackson ImmunoResearch (Pennsylvania, USA)	Cat# 711-175-152; RRID: AB_2340607
Dvl2 (30D2) Rabbit Monoclonal Antibody	Cell Signaling Technology	Cat#3224; RRID: RRID: AB_2093336
Glyceraldehyde 3-phosphate dehydrogenase (GAPDH) antibody (6C5) Mouse monoclonal antibody	Thermo Fisher Scientific	Cat# AM4300; RRID: AB_2536381
Horseradish peroxidase-conjugated Donkey anti-mouse IgG (H + L) secondary antibody	Thermo Fisher Scientific	Cat#A1601; RRID: AB_2763448
Horseradish peroxidase-conjugated Donkey anti-rabbit IgG (H + L) secondary antibody	Thermo Fisher Scientific	Cat#A1602; RRID: AB_2534697
Ki-67 AlexaFluor®647 Conjugated (B56) Mouse Monoclonal Antibody	Benedict Dickinson (BD) Biosciences	Cat#558615; RRID: AB_647130
MAPK-p44/42, Erk1/2 Rabbit Monoclonal Antibody	Cell Signaling Technology	Cat#9102; RRID: AB_330744
MET (D1C2) Rabbit Monoclonal Antibody	Cell Signaling Technology	Cat#8198; RRID: AB_10858224
NY-ESO-1 (D1Q2U) Rabbit Monoclonal Antibody	Cell Signaling Technology	Cat#45437; RRID: AB_2799282
*p*-MAPK-p44/42, Erk1/2, Thr202/Tyr204) Rabbit Monoclonal Antibody	Cell Signaling Technology	Cat#9101; RRID: AB_331646
Phospho-Cofilin (Ser3) (77G2) Rabbit Monoclonal Antibody	Cell Signaling Technology	Cat#3313; RRID: AB_2080597

**Chemicals, peptides, and recombinant proteins**		

Antibiotic-antimycotic	Thermo Fisher Scientific	Cat#15240062
B27 Supplement Minus Vitamin A	Thermo Fisher Scientific	Cat#12587
cOmplete™ EDTA-free Protease Inhibitor Cocktail Tablets	Roche Applied Science	Cat#COEDTAF-RO
Dimethyl sulfoxide (DMSO)	Sigma-Aldrich	Cat# D2653
Dulbecco’s modified eagle medium (DMEM) High Glucose Pyruvate	Thermo Fisher Scientific	Cat#11995065
DMEM/F-12	Thermo Fisher Scientific	Cat#11320033
Dulbecco’s Phosphate-Buffered Saline (DPBS)	Thermo Fisher Scientific	Cat#14190144
Ethylenediamine tetraacetic acid (ETDA)	Thermo Fisher Scientific	Cat#AM9260G
Fetal bovine serum (FBS)	Thermo Fisher Scientific	Cat#10099-141
Glucose	Sigma-Aldrich	Cat#G8769
GlutaMAX^TM^ supplement	Thermo Fisher Scientific	Cat#35050061
Heparin solution	StemCell Technologies	Cat#07980
HEPES (4-(2-hydroxyethyl)-1-piperazineethanesulfonic acid)	Thermo Fisher Scientific	Cat#15630080
Human EGF Recombinant Protein	Thermo Fisher Scientific	Cat#PHG0311
human Epidermal Growth Factor (H-EGF)	Shenandoah Biotechnologies	Cat#100-26
Human FGF-basic (FGF-2/bFGF)	Thermo Fisher Scientific	Cat#PHG0021
human Fibroblast Growth Factor (H-FGF)	Shenandoah Biotechnologies	Cat#100-146
human Platelet-derived Growth Factor AA (H-PDGF-AA)	Shenandoah Biotechnologies	Cat#100-16
human Platelet-derived Growth Factor BB (H-PDGF-BB	Shenandoah Biotechnologies	Cat#100-18
Insulin-transferrin-selenium sodium pyruvate (ITS) without vitamin A	Thermo Fisher Scientific	Cat#51300044
KnockOut™ DMEM/F-12	Thermo Fisher Scientific	Cat#12660012
Laminin	Thermo Fisher Scientific	Cat#23017015
L-Glutamine	Thermo Fisher Scientific	Cat#25030081
LDN-193189 (SMAD inhibitor)	StemCell Technologies	Cat#72147
Matrigel Basement Membrane Matrix LDEV-Free (Growth Factor Reduced)	Corning™	Cat#356230
MEM non-essential amino acids	Thermo Fisher Scientific	Cat#11140050
N2 supplement	Thermo Fisher Scientific	Cat#17502001
Neurobasal™ Medium	Thermo Fisher Scientific	Cat# 21103049
Neurobasal™-A	Thermo Fisher Scientific	Cat#10888022
Optimal Cutting Temperature (OCT) compound	Sakura Finetek	Cat#4583
Paraformaldehyde	Sigma-Aldrich	Cat#P6148
Penicillin/Streptomyocin	Thermo Fisher Scientific	Cat#15070063
Phenylmethanesulfonyl fluoride (PMSF)	Sigma-Aldrich	Cat#P7626
Polybrene infection/transfection reagent	Sigma-Aldrich	Cat#TR-1003
PTU (16-{[(4-methylphenyl)carbamoyl]amino}hexadecanoic acid)	Synthesized by Dr Tristan Rawling, University of Technology Sydney, Australia^4^	N/A
SB431542 (SMAD inhibitor)	StemCell Technologies	Cat#72234
Sodium dodecyl sulfate (SDS)	Sigma-Aldrich	Cat#71725
Sodium fluoride	Sigma-Aldrich	Cat#201154
Sodium orthovanadate	Sigma-Aldrich	Cat#S6508
Sodium pyruvate	Thermo Fisher Scientific	Cat#11360070
StemPro Neural Supplement	Thermo Fisher Scientific	Cat#A1050801
TeSR-E8 basal medium + supplement	StemCell Technologies	Cat#05990
Vitronectin XF™	StemCell Technologies	Cat#07180

**Critical commercial assays**		

Syto™ 83 nucleic acid stain	Thermo Fisher Scientific	Cat#S11364
LIVE/DEAD™ Viability/Cytotoxicity Kit	Thermo Fisher Scientific	Cat#L3224
PEG-based hydrogel BioInks	Inventia Life Science	N/A

**Deposited data**		

Original western blot images	This paper	https://doi.org/10.17632/7tb99r5f65.1

**Experimental models: Cell lines**		

H9/WA09 human embryonic stem cells (hESCs)	WiCell; Agreement No. 19-W0538	RRID: CVCL_9773
HEK293T	American Type Culture Collection	ATCC# CRL-11268; RRID: CVCL_1926
SU-DIPG-XXIV (DIPG24)	Professor Michelle Monje, Stanford University, USA^38^	RRID: CVCL_C1MY
U87MG	American Type Culture Collection	ATTC# HTB-14; RRID: CVCL_0022
WK1	Drs Brett Stringer and Bryan Day, Queensland Institute of Medical Research, Australia^24^	https://qimrberghofer.edu.au/q-cell/; RRID: CVCL_VS54

**Recombinant DNA**		

pLentipuro3/TO/V5-GW/EGFP-Firefly Luciferase	Addgene	Cat#119816
psPAX2	Addgene	Cat#12260
pMD2.G	Addgene	Cat#12259

**Software and algorithms**		

g:Profiler	Raudvere et al.^27^	https://biit.cs.ut.ee/gprofiler/gost
GeoMx™ Digital Spatial Profiler (DSP) analysis suite	NanoString™ Technologies	N/A
GraphPad Prism	GraphPad	https://www.graphpad.com/
ImageJ (Fiji)	ImageJ Open Source Software	https://imagej.net/ij/
Leica Application Suite (LAS) v.2.7.3.9723	Leica Microsystems	https://www.leica-microsystems.com/products/microscope-software/p/leica-las-x-ls/
Metamorph v7.7	Molecular Devices	N/A
Python (Anaconda Software Distribution)	Python Software Foundation/Anaconda Software Distribution	https://www.python.org

**Other**		

BD FACSAria III cell sorter	BD Biosciences	https://www.bdbiosciences.com/en-au/products/instruments/flow-cytometers/research-cell-sorters/bd-facsaria-iii
Dry block heater	Thermoline Scientific	N/A
GeoMx™ (DSP)	NanoString™ Technologies	https://brukerspatialbiology.com/products/geomx-digital-spatial-profiler/
Juli™Stage in-incubator inverted microscope	NanoEntek	N/A
Leica DMi8 inverted microscope	Leica Microsystems	https://www.leica-microsystems.com/products/light-microscopes/p/dmi8/
Leica TCS SP5 Inverted Confocal	Leica Microsystems	https://www.leica-microsystems.com/products/confocal-microscopes/p/leica-tcs-sp5/
NuPAGE® Novex 4–12% Bis-Tris mini gels	Thermo Fisher Scientific	Cat#NP0322BOX
Personal Vortex mixer	Ratek	N/A
Short Tandem Repeat (STR) profiling	Cell Bank Australia	https://cellbankaustralia.com/
Stem cell karyotyping	Sullivan Nicolaides Pathology (via Sonic Genetics)	https://www.sonicgenetics.com.au/our-tests/all-our-tests/karyotype-blood/
Ultra-low attachment (ULA), U-bottom 96-well plates	Corning™	Cat#CLS4520
Ultra-low attachment (ULA), 6-well plates	Corning™	Cat#CLS3471
Zeiss Axio Observer inverted epifluorescence microscope	Zeiss	https://www.zeiss.com/microscopy/en/products/light-microscopes/widefield-microscopes/axio-observer-for-life-science-research.html

### Experimental model and study participant details

#### Cell culture

Primary, patient-derived adult glioblastoma (GBM) cell line WK1 (male, age 77 years), supplied by Stringer, Day and Boyd^39^ was cultured as previously described^40^ for a maximum of 35 passages. Briefly, WK1 cells were maintained in filter-capped tissue culture flasks coated with Matrigel solution (1% Matrigel growth factor reduced Basement Membrane Matrix LDEV-Free (v/v), DMEM High Glucose Pyruvate). Cell media (GNS media) consisted of KnockOut DMEM/F-12 supplemented with human EGF recombinant protein (20 ng/mL), human FGFb (10 ng/mL), Glutamine (2 mM), Heparin (20 ng/mL), Penicillin/Streptomyocin (100 U/mL) and StemPro Neural Supplement (1X concentration). Cells were maintained at 37 °C and 5% CO_2_.

Primary, patient-derived Diffuse Intrinsic Pontine Glioma (DIPG)/Diffuse Midline Glioma (DMG) cell line SU-DIPG-XXIV (DIPG24; female, age 6 years; supplied by Professor Monje^41^) was cultured in Tumor Stem Media (TSM): a 1:1 mixture of Neurobasal-A and DMEM/F-12, supplemented with B27 Supplement Minus Vitamin A (1:50), 20 ng/mL human Epidermal Growth Factor (H-EGF), 20 ng/mL human Fibroblast Growth Factor (H-FGF), 10 ng/mL human Platelet-derived Growth Factor AA (H-PDGF-AA), 10 ng/mL human Platelet-derived Growth Factor BB (H-PDGF-BB), 2 μg/mL heparin, 1% antibiotic-antimycotic, 1x GlutaMAX, 10 mM HEPES, 1x MEM Non-Essential Amino Acids Solution and 1 mM sodium pyruvate. Cells were maintained at 37°C, 5% CO_2_ as neurospheres.

U87MG GBM cells (male, 44 years; obtained from American Type Culture Collection: catalog number HTB-14) were maintained as adherent cultures in DMEM supplemented with 10% (v/v) fetal bovine serum (FBS) at 37°C, 5% CO_2_. HEK293T cells (female, embryonic; ATCC# CRL-11268) were cultured in DMEM supplemented with 10% FBS and 1% MEM non-essential amino acids.

H9/WA09 human embryonic stem cells (H9 hESCs; female, embryonic; Agreement No. 19-W0538; WiCell, USA) were cultured as previously described^21^ in feeder-free conditions on vitronectin-coated plates using TeSR-E8 basal medium plus supplement. Stem cells were karyotyped to confirm genomic stability (Sullivan Nicolaides Pathology, Brisbane, Australia), and no abnormalities were detected.

The study was not designed to evaluate sex-dependent responses, and the limited number of patient-derived cell lines precludes meaningful assessment of sex-specific effects.

##### Ethics

All stem cell culture and procedures were approved and carried out in accordance with guidelines of the Sydney Children’s Hospital Network Human Research and Ethics Committee (2019/ETH00240). Research involving H9 human embryonic stem cells in this study, comprising routine culture and maintenance, directed differentiation into cortical organoids, and assembloid co-culture, falls under Category 1a of the ISSCR, Guidelines for Stem Cell Research and Clinical Translation (2021),^42^ which encompasses research permissible without specialist oversight review. H9 hESCs (WA09) were obtained from WiCell Research Institute under a Material Transfer Agreement (Agreement No. 19-W0538). No genetic modification of hESCs was performed in this study.

All cell line identities were independently verified by short tandem repeat (STR) profiling (Cell Bank Australia), and all cultures are routinely tested for mycoplasma contamination.

### Method details

#### Western blot analysis

Cells were lysed in 3% SDS lysis buffer containing phosphatase and protease inhibitors (50 mM sodium fluoride, 1 mM sodium orthovanadate, 1 mM PMSF, and EDTA-free Protease Inhibitor Cocktail Tablets from Roche Applied Science). Twenty μg total protein extracts were resolved by SDS-PAGE on NuPAGE Novex 4–12% Bis-Tris mini gels (Thermo Fisher Scientific, VIC, AU). Phospho-Cofilin (Ser3) (77G2) Rabbit Monoclonal Antibody (#3313, 1:1000), Cofilin (D3F9) Rabbit Monoclonal Antibody (#5175, 1:1000) and Dvl2 (30D2) Rabbit Monoclonal Antibody (#3224, 1:1000) were purchased from Cell Signaling Technology (Danvers, MA). Anti-Dishevelled 2 (phospho S143) antibody (EPNCIR145, ab124933, 1:500) was from Abcam (AU). Mouse monoclonal glyceraldehyde 3-phosphate dehydrogenase (GAPDH) antibody (6C5, 1:10,000, Thermo Fisher Scientific, VIC, AU) was used as loading control in Western blot analyses.

#### Lentivirus production using HEK293T cells

HEK293T cells were seeded at 4 × 10^6^ cells per 10 cm tissue culture plate ∼20 h prior to transfection. On the next day, media was removed and cells were cultured in 10 mL fresh OptiPro SFM media (OPS, supplemented with 1x GlutaMAX) containing 25 μM chloroquine diphosphate (Sigma, C6628) in incubator for 5 h. Plasmids mixture was prepared in one tube with 500 μL OPS media including pLentipuro3/TO/V5-GW/EGFP-Firefly Luciferase (Addgene; Cat no. 119816), packaging (psPAX2; Addgene #12260) and envelope (pMD2.G; Addgene #12259) vectors. Another 500 μL OptiPro SFM containing Polyethylenimine “MAX”(linear MW 40,000 Da, 1 mg/mL; Polysciences Cat No 24765-1) (ratio of DNA (μg): PEI (μg) = 1:3) was added dropwise to the plasmids solution and incubated for ∼20 min at RT to form transfection complex. The transfection complex was carefully transferred to the HEK 293T packaging cells without dislodging the cells. After media change on the following day, virus-containing media were harvested at 48 h and 72 h post transduction and stored at 4°C. Combined media were centrifuged at ∼500*g* for 5 min and the supernatant was filtered through a 0.45-μm PES filter. Apply the filtered culture supernatant to Vivaspin (Turbo 15; Sartorius, Cat No VS15T41) and centrifuge at 3000*g* for 20 min at 16°C. Collect the remaining lentiviral particles from the tube and store aliquots sto at −80°C. Virus functional titers (TU/mL) were determined by flow cytometry analysis (BD FACSCanto II System, BD Biosciences) of GFP positivity in transduced HEK293T cells.

#### Generation of EGFP-luciferase cell lines

WK1 and DIPG24 cells were seeded at 40,000 cells per well in 12-well plate coated with Matrigel solution 24 h prior to transduction. On the next day, LV particles were added (multiplicity of infection = 5) with 10 μg/mL polybrene (hexadimethrine bromide, Sigma#H9268) in fresh complete GNS media. Virus containing media were removed after 16 h and at 96 h post transduction, puromycin selection (0.5 μg/mL) was conducted for 10 days to generate stable clones. Alternatively, GFP positive cells were sorted using a BD FACSAria III cell sorter (BD Biosciences).

#### PTU

The ureido fatty acid analogue PTU (16-{[(4-methylphenyl)carbamoyl]aminohexadecanoic acid) was prepared as reported previously.^4^ Before each use, stock solutions were heated to ∼80°C in a dry block heater (Thermoline Scientific, Australia) for 10 s and vortexed (Ratek, Australia) until the solution appeared clear. Cells were treated with 10 μM PTU or Dimethyl sulfoxide (DMSO; Sigma#D2653) vehicle control for 72 h unless otherwise indicated.

#### MCTS, cortical organoids and assembloids

MCTS were formed by seeding cells in ultra-low attachment (ULA), U-bottom 96-well plates (Corning, USA), 15000 and 5000 cells per well for WK1 and DIPG24 cell lines, respectively. Spheroids aggregated over 3–5 days.

Cortical organoids were generated from H9 hESCs using a directed protocol as previously described.^21^ Briefly, H9 hESCs (∼30,000 cells/well) was seeded into Laminin-coated 6-well plate and culture for 24 h with replaced with Neural induction medium (NIM) (Neurobasal Medium, DMEM/F-12, 1x N2 supplement, 1x B27 without vitamin A supplement, 1x ITS (Insulin-Transferrin-Selenium) without vitamin A, 2 mM GlutaMAX, 0.3% (w/v) glucose, supplemented with dual SMAD inhibitors (0.1 μM LDN193189, 10 μM SB431524). Media was replenished every 24–48 h until neural rosettes formed on top of colonies (<6 days) to be harvested using 0.5 mM EDTA. Harvested neural progenitor cells (NPC) were centrifuged at 200*g* for 3 min, and resuspended in ‘Neural Media’ (NM) consisting of Neurobasal Medium, 1x N2 Supplement, 1x B27 without vitamin A supplement, 1x ITS without vitamin A, 2 mM GlutaMAX, and supplemented with 20 ng/mL EGF and 20 ng/mL FGF2, and transferred to ULA U-bottom 96-well plates to form neuropsheres. Neurospheres were matured for 14 days with half media change every 3^rd^ day before moving to a ULA 24- or 6-well plate for subsequent culture in an orbital shaker incubator at 85 RPM.

Assembloids were formed by combining organoids (∼3 weeks post induction) and MCTS, 1:1, in each well of a ULA U-bottom 96-well plate.^21^ Assembloids were allowed to form over a 24 h period, after which time PTU was introduced to the cultures which were then incubated for a further 3 days. Co-cultures were monitored with a DMi8 Inverted microscope (Leica Biosystems, Germany) and Leica Application Suite (LAS) v.2.7.3.9723 software was used to generate tile-stitched images.

#### Invasion

MCTS were embedded^40^ in Matrigel hydrogels for invasion analysis. Individual MCTS were collected and washed in Dulbecco’s phosphate-buffered saline (DPBS, Thermo Fisher Scientific) prior to embedding in undiluted Matrigel in 96-well plates. Media ± treatment was added directly to MCTS after 1 h of Matrigel polymerisation at 37°C, followed by time-lapse imaging every 20 min for 72 h with an in-incubator inverted microscope (JuliStage, (NanoEntek, South Korea), 4× magnification with an UPLANFL objective (bright-field illumination). Invasion fronts were determined by digitally excluding the dense central mass (core) of cells through image segmentation at the first time point. The area of invasion was then quantified from each MCTS at 72 h using Metamorph v7.7 software (Molecular Devices, USA).

#### Bioprinting

Cells were bioprinted with a Rastrum 3D bioprinter (Inventia Life Science, Australia) as previously described.^25^ Commercially purchased polyethene glycol (PEG)-based “bioinks” and crosslinking activators (Inventia Life Science, Australia) were used to generate hydrogels with defined elastic moduli. Where indicated, hydrogels were biofunctionalised with the adhesion peptide motif arginine-glycine-aspartate (RGD). Live/dead staining of bioprinted cells (ThermoFisher Scientific, USA) was performed as per the manufacturer’s guidelines.

#### Semi-automated 3D single-cell tracking

Single-cell tracking assays (∼100 cells per well) were performed on time lapse images of bioprinted HGG cells (20 min intervals for 72 h) collected using a Zeiss Axio Observer inverted epifluorescence microscope (Zeiss, Germany) at 37°C, 5% CO_2_ (at 10× with a Plan-Apochromat objective and bright-field illumination). Semi-automated cell tracking was achieved using the “Track Objects” function in Fiji. Poorly-illuminated, dividing or apoptotic cells were excluded from analysis. Average speed and mean squared displacement were calculated as previously described^40^ using Excel and Python (Python Software Foundation, USA) software, respectively. Python scripts to automate the calculation of MSD data were kindly generated by Ms Weijie Chen.

#### Immunofluorescence

Similar to previously described experiments,^21^ assembloids were fixed with 4% (w/v) PFA solution at 4°C for overnight and then washed with PBS before incubated in 20% (v/v) sucrose solution at RT for 6–8 h. Samples were mounted in Cryomolds (Sakura Finetek, USA) using optimal cutting temperature (OCT) compound and frozen on dry ice before stored at −80°C. Samples were sectioned at 15 μm using a CM1950 cryostat (Leica Biosystems, Germany), and placed on Superfrost slides (Thermo Fisher Scientific, USA). Slides were air-dried overnight and then stored at −20°C before analyzed by immunofluorescence. Details of antibodies can be found in the key resources table. Sections were imaged with a Leica TSC SP5 Inverted Confocal System (Leica Biosystems, Germany) with 488 nm-argon, 405 nm-diode, 543 nm-HeNE and 633 nm-HeNE lasers set to 20% power. Image resolution was fixed at 2048 × 2048 pixels, line average settings were set to 4, and all images were acquired with a z-step of 1–3 μm. Gain and offset were adjusted per sample and maintained per image replicate. Images were acquired with HCX PL APO CS oil immersion 20×, 40× or 63× objectives (Leica Biosystems, Germany). Fluorescence intensity was quantified using Corrected Total Cell Fluorescence in ImageJ (Fiji) (integrated density – (Area × Mean background fluorescence). Integrated density and area were measured for each marker protein and background intensity was measure from a cell-free region of the image. Where indicated Z-stacks of confocal slices were merged into maximum-intensity projections for analysis.

#### GeoMx spatial profiling and data analyses

Assembloid cryosections (5 μm) were shipped frozen to NanoString Technologies in San Diego, USA for analysis as per established NanoString GeoMx Digital Spatial Profiling (DSP) protein assay protocols. Morphology markers included Syto 83 nucleic acid stain (ThermoFisher Scientific, USA) and Vimentin. Regions of interest (ROIs) were selected by delineating GFP-tagged MCTS. Tumor cells were segmented by masking GFP-positive cells and nuclei. The immediately adjacent section was designated peri-tumoural (PT). Tissue adjacent to the PT was defined as proximal, and the opposite side of the MCTS was defined as distal.

#### DSP data analysis

The nCounter technical variability and spontaneous hybridization was controlled for by use of a group of sequences not homologous to any known organism, termed the External RNA Control Consortium (ERCC). Sequences were added to the protein assay downstream of oligo collection. The ERCC normalised dataset was normalised against the housekeeper proteins Histone H3 and ribosomal protein S6, and signal to noise ratios (SNR) were normalised against IgG isotype controls mouse IgG1, mouse IgG2a and rabbit IgG. Area normalisation of the geometric mean of ROIs was also conducted to account for differences in cellularity across regions. Principal component analysis (PCA) and principal component regression of the final normalised datasets was performed with GraphPad Prism.

Differential protein expression (DE) data were generated using a Linear Mixed Model (LMM) with Benjamini-Hochberg (BH) correction within the GeoMx DSP analysis suite, including adjustment to random effects between co-culture sections on each slide. For within-slide comparisons (ROI versus ROI), multiple levels (ROIs) within the random effect factor (sections) were accounted for with the application of a random slope correction. Statistical significance of DE was determined by an adjusted *p*-value <0.05, calculated as the antilog of −log10 adjusted *p*-values using Excel software. Clustered hierarchical dendrograms were generated within the DSP analysis suite using a custom R-script “General Heatmap DSP” (Nanostring, USA), while volcano plots from DE data were constructed with Python (Anaconda Software Distribution [https://anaconda.com]). Further enrichment analysis was performed with G-Profiler {[https://biit.cs.ut.ee/gprofiler/gost].^38^

### Quantification and statistical analysis

Statistical analyses were performed in GraphPad Prism, except for the spatial profiling data, which were analyzed within the GeoMx DSP analysis suite as detailed above. The specific statistical test, definition and value of n, and dispersion measure (mean ± SEM) for each experiment are reported in the corresponding figure legend and in the relevant method details subsection. Significance was defined as *p* < 0.05, with significance levels indicated in the figures (∗*p* < 0.05, ∗∗*p* < 0.01, ∗∗∗*p* < 0.001, ∗∗∗∗*p* < 0.0001).
