# Supplementary material for: Brain assembloids reveal efficacy and off target effects in high grade glioma brain cancer
Source: iScience. 2026 Aug 19;29(9):117247. doi: 10.1016/j.isci.2026.117247 (PMC13521100; doi:10.1016/j.isci.2026.117247)
Supplement: Supplementary file 1 [file mmc1.pdf]

iScience, Volume *29*

Supplemental information

Brain assembloids reveal efficacy and off target effects in high grade glioma brain cancer

Farhana Amy Sarker, Yuyan Chen, Victoria Prior, Sam Bax, Louise Orcheston-Findlay, Tom Grundy, Thuvarahan Jegathees, Robert Utama, Martin Engel, Tristan Rawling, Yassir Al Zubaidi, Michael Murray, and Geraldine Margaret O'Neill

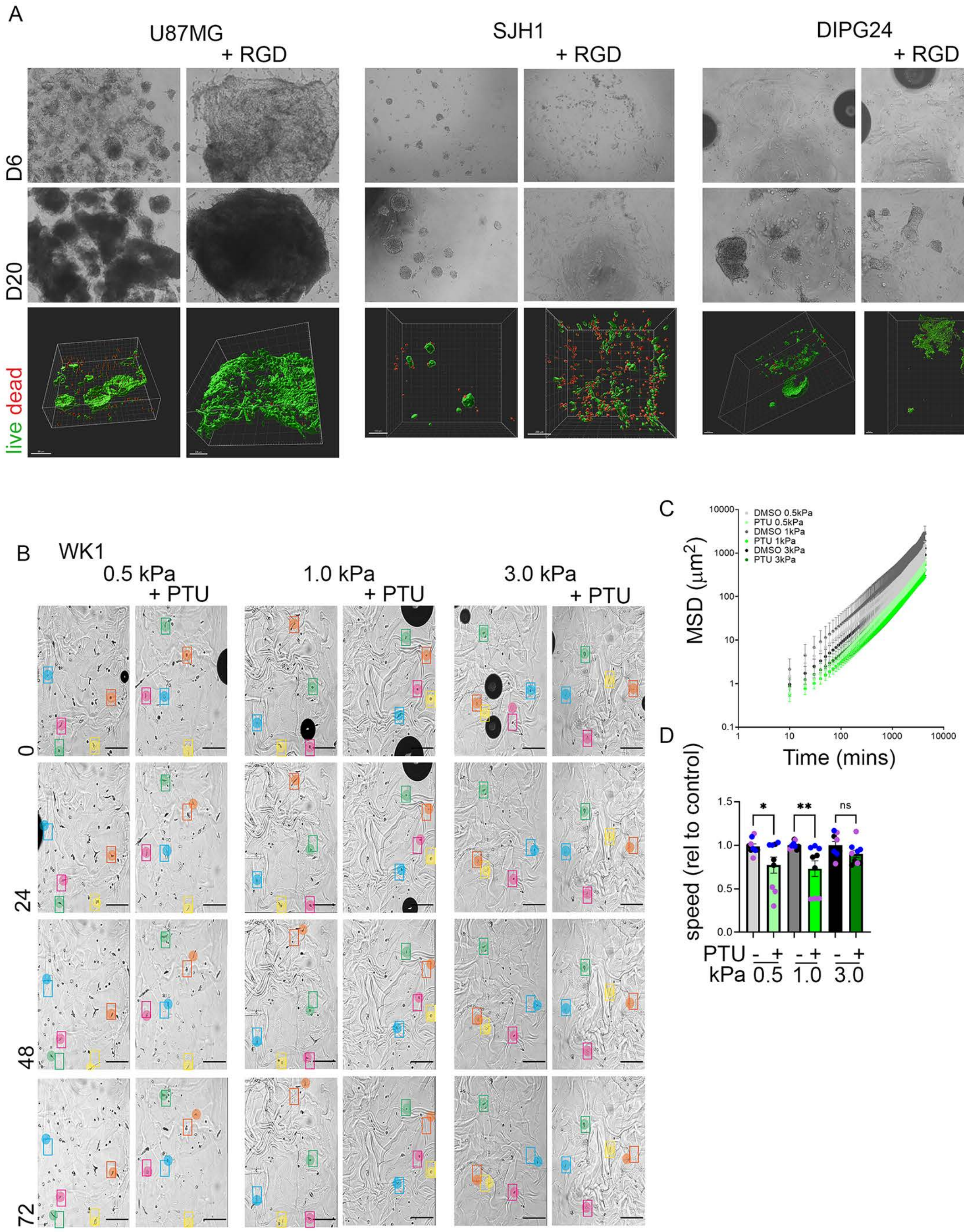

Supplementary Figure 1. Exploratory analysis of stiffness-dependent invasion and PTU response in a bioprinted model. A. Comparison of HGG lines bioprinted in uncoupled and RGD-coupled PEG hydrogels (BioInksTM), 6 days and 20 days after printing. Bottom row shows 3D renderings of confocal z-stacks of cultures after 20 days, stained for live (green) and dead (red) cells. B. Bright-field images of low density WK1 cells bioprinted in RGD-coupled BioInksTM formulated to create gels with 0.5 kPa, 1 kPa and 3 kPa Young's moduli. Following addition of drug, time-lapse images were captured over a 24-hour period. Coloured boxes around cells at 0h delineate the cells' starting positions.Tracked cells are highlighted in the same colour, showing cell displacement over time. Scale bars = 100 μm. C. Mean Squared Displacement (MSD) plots showing WK1 cell displacements over the 72-hour time-lapse period in gels of 0.5, 1.0 and 3 kPa. Control data (grey) and PTU treated cells (green). X and Y axes are Log10 transformed. D. Cell speeds relative to DMSO controls. Coloured data points = 3 independent print runs, with 3 technical repeats per print run, average 53 ± 4 (SEM) cells tracked per technical repeat (minimum 17, maximum 139 cells in individual replicates). $*p < 0.05$, $** p < 0.01$, NS = not significant, Students' t-test.

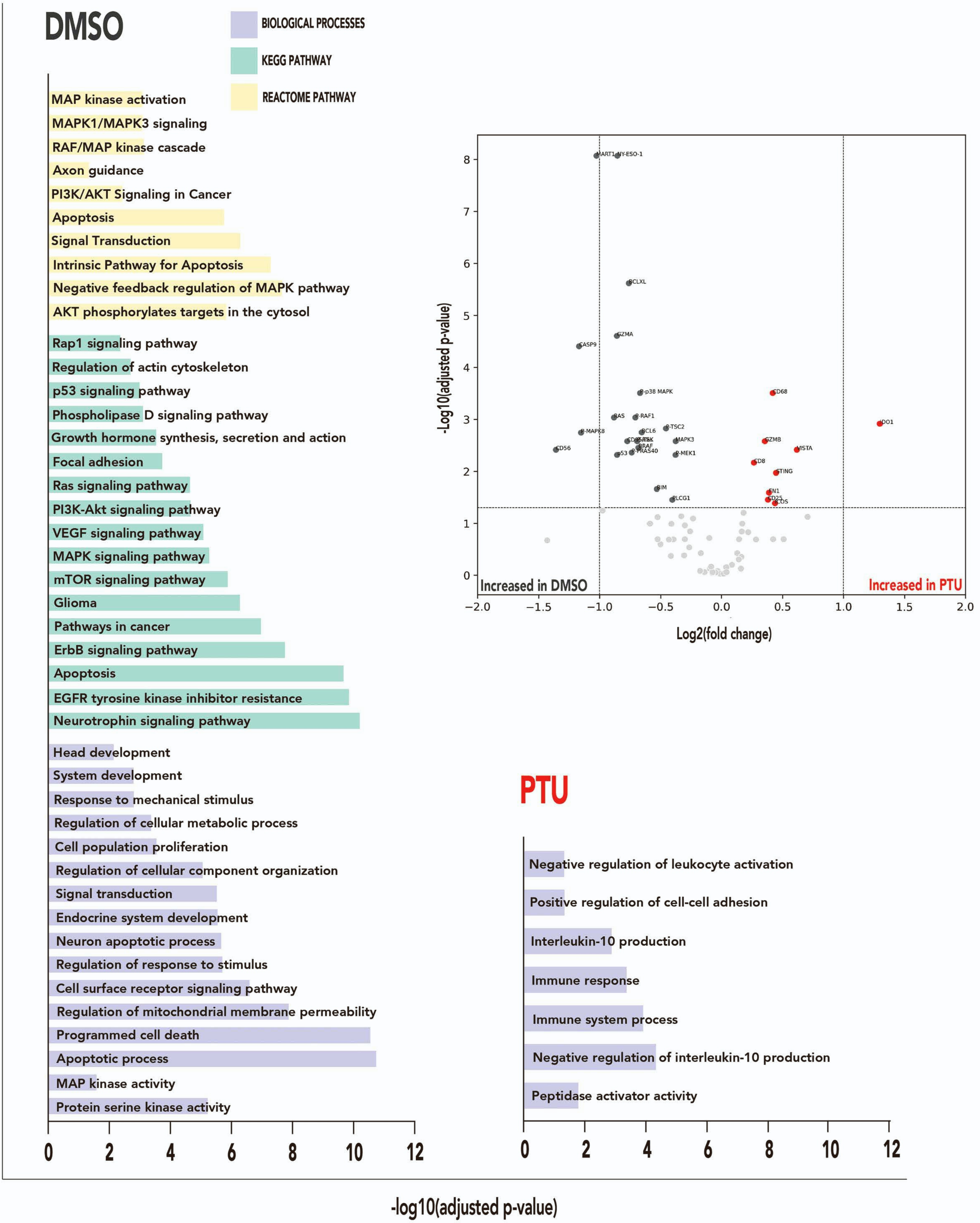

Supplementary Figure 2. PTU treatment reduces expression of cancer related signaling pathways in assembloids. Volcano plots show differential protein expression, Log2 (fold change) of DMSO versus PTU-treated assembloids. Indicated proteins are significantly overexpressed (p-adjusted < 0.05) in either DMSO (black) or PTU (red)-treated assembloids. Bar charts represent outcomes of GO enrichment analysis of the significantly different proteins and are grouped based on enrichment strategy (indicated in key). Significance is indicated on the x-axis (-log10 (adjusted p-value)).

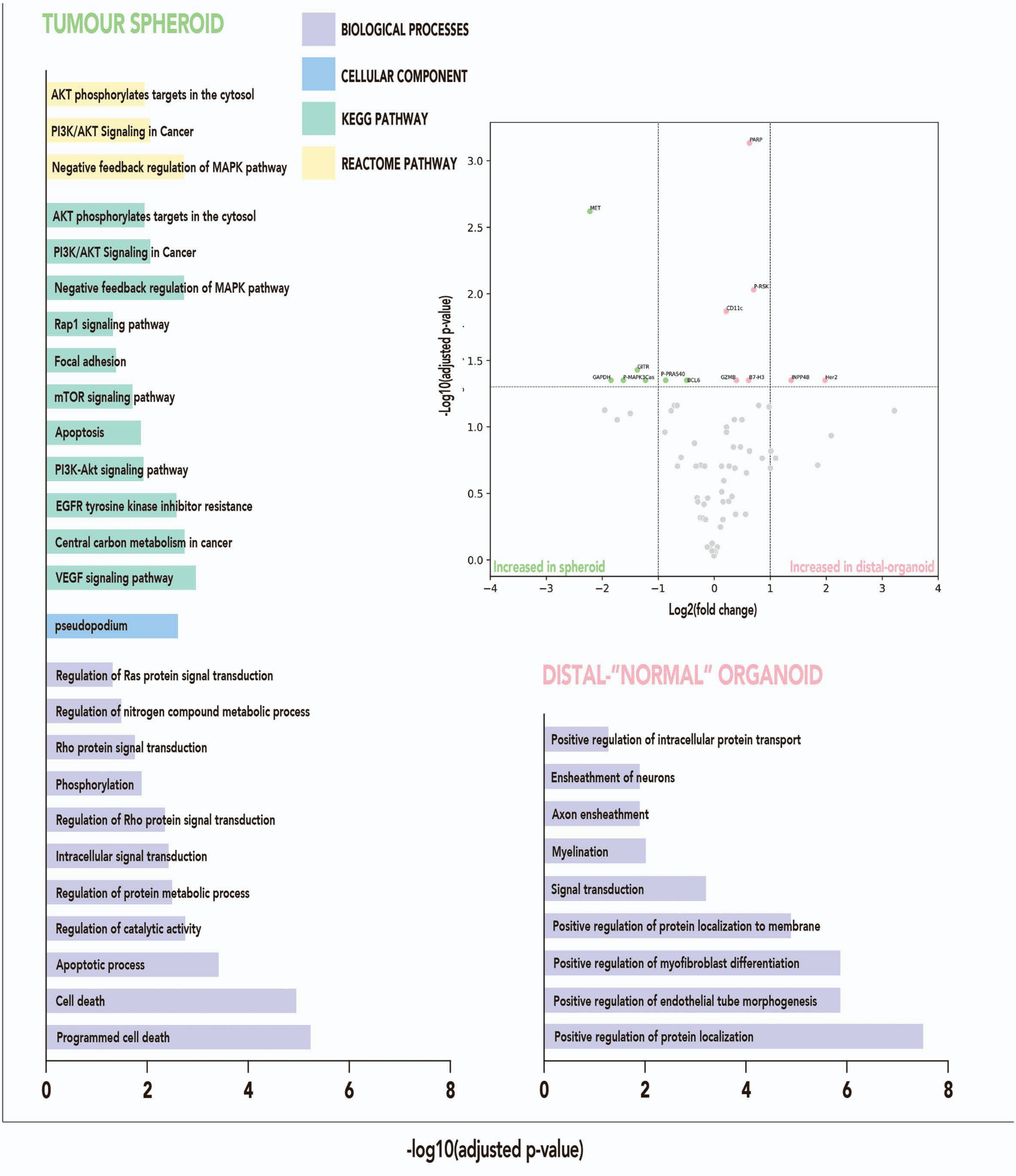

Supplementary Figure 3. Differential protein expression between MCTS and cortical organoids in assembloid cultures. Volcano plots show differential protein expression, Log2 (fold change) of TS vs distal-organoid ROIs in control assembloids. Indicated proteins are significantly overexpressed (p-adjusted < 0.05) in either TS (green) or distal organoids (pink). Bar charts represent outcomes of GO enrichment analysis of the significantly different proteins and are grouped based on enrichment strategy (indicated in key). Significance is indicated on the x-axis (-log10 (adjusted p-value)).
